# Novel Intraoperative Imaging of Gastric Tube Perfusion during Oncologic Esophagectomy—A Pilot Study Comparing Hyperspectral Imaging (HSI) and Fluorescence Imaging (FI) with Indocyanine Green (ICG)

**DOI:** 10.3390/cancers14010097

**Published:** 2021-12-25

**Authors:** Sebastian Hennig, Boris Jansen-Winkeln, Hannes Köhler, Luise Knospe, Claire Chalopin, Marianne Maktabi, Annekatrin Pfahl, Jana Hoffmann, Stefan Kwast, Ines Gockel, Yusef Moulla

**Affiliations:** 1 Department of Visceral, Transplant, Thoracic and Vascular Surgery, University Hospital of Leipzig, Liebigstr. 20, D-04103 Leipzig, Germany; Sebastian.Hennig@medizin.uni-leipzig.de (S.H.); Boris.Jansen-Winkeln@sanktgeorg.de (B.J.-W.); Marie.Knospe@medizin.uni-leipzig.de (L.K.); 2 Department of General, Visceral, Thoracic and Vascular Surgery, St. Georg Hospital, Delitzscher Str. 141, D-04129 Leipzig, Germany; 3 Innovation Center Computer Assisted Surgery (ICCAS), University of Leipzig, Semmelweisstr. 14, D-04103 Leipzig, Germany; hannes.koehler@medizin.uni-leipzig.de (H.K.); claire.chalopin@medizin.uni-leipzig.de (C.C.); Marianne.Maktabi@medizin.uni-leipzig.de (M.M.); Annekatrin.Pfahl@medizin.uni-leipzig.de (A.P.); 4 Department of Sports Medicine and Prevention, University Leipzig, Rosa Luxemburg Str. 20-30, D-04103 Leipzig, Germany; Jana.hoffmann@uni-leipzig.de (J.H.); stefan.kwast@uni-leipzig.de (S.K.)

**Keywords:** hyperspectral imaging (HSI), fluorescence imaging (FI), indocyanine green (ICG), anastomotic leak (AL), Ivor Lewis esophagectomy, gastric conduit perfusion

## Abstract

**Simple Summary:**

Oncologic esophagectomy with gastric conduit reconstruction is the gold standard in the curative treatment of localized esophageal cancer. Anastomotic leakage is one of the most significant postoperative complications and a predictor of increased postoperative mortality and deteriorated quality of life. Adequate perfusion is one of the essential prerequisites for anastomotic healing. An objective evaluation of the perfusion of the gastric conduit can be performed by hyperspectral imaging (HSI) and fluorescence imaging (FI) with indocyanine green (ICG) intraoperatively. The aim of this pilot study was to evaluate the feasibility and the potential of improved outcomes by simultaneous HSI and FI-ICG measurements of the gastric tube during esophagectomy.

**Abstract:**

Background: Novel intraoperative imaging techniques, namely, hyperspectral (HSI) and fluorescence imaging (FI), are promising with respect to reducing severe postoperative complications, thus increasing patient safety. Both tools have already been used to evaluate perfusion of the gastric conduit after esophagectomy and before anastomosis. To our knowledge, this is the first study evaluating both modalities simultaneously during esophagectomy. Methods: In our pilot study, 13 patients, who underwent Ivor Lewis esophagectomy and gastric conduit reconstruction, were analyzed prospectively. HSI and FI were recorded before establishing the anastomosis in order to determine its optimum position. Results: No anastomotic leak occurred during this pilot study. In five patients, the imaging methods resulted in a more peripheral adaptation of the anastomosis. There were no significant differences between the two imaging tools, and no adverse events due to the imaging methods or indocyanine green (ICG) injection occurred. Conclusions: Simultaneous intraoperative application of both modalities was feasible and not time consuming. They are complementary with regard to the ideal anastomotic position and may contribute to better surgical outcomes. The impact of their simultaneous application will be proven in consecutive prospective trials with a large patient cohort.

## 1. Introduction

Oncologic esophagectomy is the cornerstone treatment of locally or locally advanced esophageal cancer. Reconstruction with a gastric conduit is widely used following oncologic resection. Anastomotic leakage (AL) of the esophago-gastrostomy is a relevant postoperative complication that represents a poor prognostic factor in patients undergoing esophagectomy [1,2]. The reported incidence of AL is 5–20%, with mortality rates up to 50% [3,4,5,6,7,8]. Considering that adequate perfusion is crucial for successful anastomotic healing [9], a precise and objective intraoperative evaluation of the anastomotic perfusion is of utmost interest. In previous studies and clinical practice, evaluation of gastric conduit perfusion has been assessed on the surgeons’ subjective experience only, a controversial issue that has been discussed [10,11]. Methods such as pulse oximetry, Doppler ultrasound, and infrared imaging have already been described to evaluate intestinal perfusion. However, none of them have consequently been integrated into the clinical routine [10]. Recently, fluorescence imaging (FI) with indocyanine green (ICG) has gained popularity in perfusion evaluation, and studies have proven its reliability also in esophageal surgery [12,13,14]. Despite its feasibility and reliability, FI has some downsides. Due to allergic reactions to ICG, it can cause anaphylactic shock in rare cases, as well as cardiovascular reactions, dyspnea, or urticaria [15,16]. Furthermore, the application of ICG generates significant costs, while it has a half-life of 3–4 min only. In addition, it can lead to interference with the following measurements. Hyperspectral imaging (HSI), in contrast, is a non-invasive imaging method that has already demonstrated its potential for perfusion assessment in clinical practice [17,18,19,20,21]. The aim of this study was to compare the results of HSI and FI against subjective evaluations in gastric conduit perfusion and determine the “ideal” anastomotic position intraoperatively.

## 2. Methods and Material

### 2.1. Study Design

This is a single-institution, one-arm prospective pilot observational study designed in accordance with the Declaration of Helsinki. The trial was registered at ClinicalTrials.gov (NCT: 04226781) and approved by the local ethics committee of the Faculty of Medicine of the University of Leipzig (026/18-EK).

### 2.2. Study Population

In this pilot study, 13 consecutive patients with oncologic esophageal resection and gastric conduit reconstruction due to esophageal cancer were included. Among 18 eligible patients, one had to be excluded due to peritoneal metastasis (progression during neoadjuvant chemotherapy), and another one was excluded because of hemodynamic instability during the operation (injection of ICG was considered as a risk). A third patient had hyperthyroidism, and thus fulfilled the criteria for exclusion. In one patient, ICG measurement had to be repeated three times and he was therefore excluded. In another patient, the ICG file was damaged due to unknown reasons. Finally, 13 patients fulfilled the criteria to participate. The preoperative findings are presented in Table 1.

### 2.3. Surgical Procedure

Each patient underwent a two-staged hybrid Ivor Lewis esophagectomy (Table 2). The operation was carried out by only two highly experienced upper gastrointestinal (UGI) surgeons (I.G. and Y.M.). At the first abdominal stage, the laparoscopic/robotic gastrolysis with abdominal D2-lymphadenectomy was performed in terms of ischemic preconditioning, three to six days before transthoracic esophagectomy [22,23]. The second stage consisted of re-laparoscopy with gastric conduit formation (width of 3.5–4 cm), right-thoracic partial esophagectomy with intrathoracic/mediastinal lymphadenectomy, and posterior mediastinal route gastric tube pull-up. Through the anterior lateral mini-thoracotomy, the surgeon marked the site of the future anastomosis with a blue line (Skin marker, Symmetry Surgical GmbH, Tuttlingen, Germany) at the peripheral end of the gastro-epiploic vessel arcade, according to subjective assessment. Afterwards, a hyperspectral record (Diaspective Vision GmbH, Am Salzhaff, Germany) of the gastric tube tip—the region of interest (ROI)—was acquired. Then, FI with ICG was performed and a video was recorded of the same region with the VisionSense Camera (Medtronic GmbH, Meerbusch, Germany). The anesthesiologists assisted with the injection of the dye in a standardized manner and dosage (peripheral i.v. application). The site of the future anastomosis was adapted according to the imaging in the case of insufficient perfusion of the gastric tube tip. If the results differed, the more central measurement result of the gastric tube was chosen as the anastomotic site. After its definition, the reconstruction between the remaining esophagus and the gastric conduit was carried out with a circular stapler (EndoGIA, Medtronic, Minneapolis, MN, USA) (28 or 25 mm in diameter, end–to–side). A tension-free anastomosis was intended for all patients. Finally, the anastomosis was covered with an omental flap, which had been constructed during the abdominal phase of the operation (minimally invasive gastrolysis).

### 2.4. Hyperspectral Imaging (HSI)

**Figure 1:**
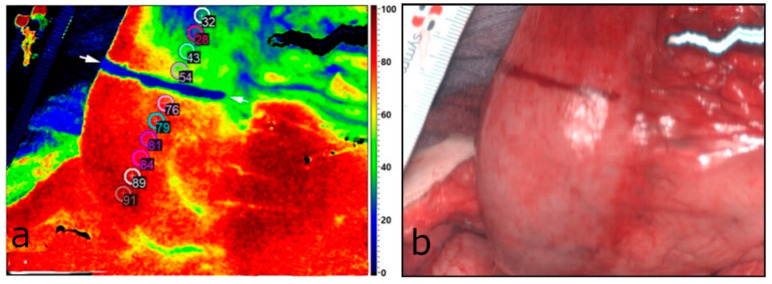
(**a**) StO_2_ parameter image of the gastric tube (lower part—central; upper part—peripheral). The marked line is visible in blue and the 10 markers with a diameter of 4 mm are placed along the gastric tube. The four markers distal to the blue line show StO_2_ < 75%. The new resection line is marked by two white arrows. In this case, it is equal to the subjective line (blue). (**b**) RGB color image of the same area.

A TIVITA^®^ Tissue hyperspectral camera system (Diaspective Vision GmbH, Am Salzhaff, Germany) was used for acquiring and analyzing the HSI data. This camera provides spectral information from 500 to 1000 nm within a field of view (FOV) of 8 × 6.5 cm^2^ and a theoretical spatial solution of 0.13 mm/pixel. The measurement takes approximately eight seconds. The system illuminates the area of interest with six halogen spotlights, at a distance of 50 cm. A push broom camera spectrograph with an integrated image sensor records the spatial dimension on the *X* axis and the spectral dimension simultaneously. The second spatial dimension along the *Y* axis is acquired by scanning with a stepper motor inside the camera housing [20]. Afterwards, the software generates a three-dimensional hyperspectral cube, where the *X* and *Y* axes represent spatial information, and the *Z* axis the specific spectral wavelengths. With this information, the system calculates one red–green–blue (RGB) color image (Figure 1b) and four, special, false-color images. These images, namely, StO_2_ (oxygen saturation) (Figure 1a), NIR-PI (near-infrared perfusion index), OHI (organ hemoglobin index), and TWI (tissue water index), provide quantitative information about oxygenation, perfusion, hemoglobin content, and tissue water. StO_2_ is presented in %, and the other indices are presented in arbitrary units. Exact calculations can be identified in Holmer et al. [24]. With the help of these values, it is possible to estimate the perfusion–related parameters [20]. Tissue oxygenation in superficial layers can be assessed. The NIR-PI provides information on oxygenation in deeper layers up to 6 mm. The amount of hemoglobin in the microcirculation can be determined with the OHI [25]. The ambient light was switched off during all measurements in order to provide standardized conditions for all measurements.

### 2.5. Fluorescence Imaging (FI) with Indocyanine Green (ICG)

**Figure 2:**
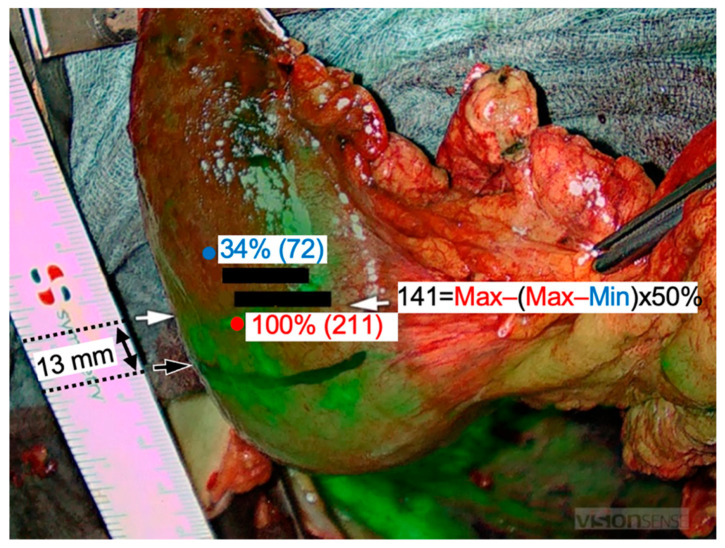
Fluorescence imaging (FI) with indocyanine green (ICG). The maximum ICG signal was 211 (red marker) and the minimum was 72 (blue marker). The subjective transection line was drawn in black intraoperatively. The ICG-based resection line was obtained 13 mm peripheral to this line at a 50% decrease of the maximum ICG signal (white arrows).

For FI, the VisionSense Iridium 3 system (Medtronic GmbH, Meerbusch, Germany) was used. It excites the fluorophore (ICG) via NIR light at 820 nm with a penetration depth of 6 mm [26]. However, the dye starts fluorescing due to modified energetic status [27]. The fluorescent signal is captured by specific scopes and cameras and can be transmitted to the original video monitor (1920 × 1080 pixels) additionally or to a standard operating room monitor. These monitors show the overlay of the RGB picture with the ICG fluorescence signal in real time (Figure 2). Measurements are possible in an open or laparoscopic setting. The intensity in the black/white image ranges from 0 to 255 (8-bit, where 0 is black and corresponds to no ICG detection, while 255 is white corresponding to full intensity of ICG fluorescence). The ICG dye is a water-soluble, non-toxic powder. For injection purposes, 25 mg of ICG was dissolved in water for injection purposes and administrated intravenously (peripheral vein). After injection, 98% of the ICG molecules bind to plasma proteins and distribute homogeneously in the vascular system, [14,28]. The dye is excreted via the hepatic system with an estimated half-life of 3–5 min [29]. The dosage of ICG was 0.1 mg/kg body weight standardized in all included patients. The cost of 25 mg ICG is about €70.

### 2.6. Postoperative Analysis

**Figure 3:**
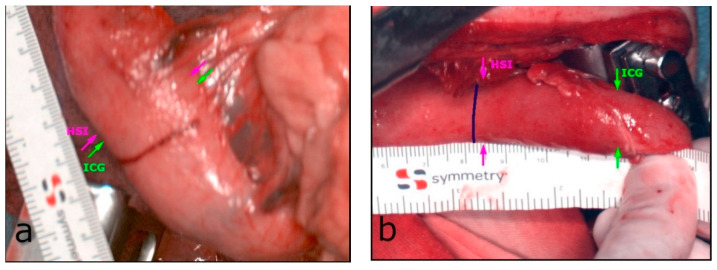
(**a**) The minimum difference between HSI (magenta arrow) and FI-ICG (green arrow), with 2.5 mm. (**b**) The maximum difference during this pilot study, with 33.5 mm.

The TIVITA^®^ Suite software (Diaspective Vision GmbH, Am Salzhaff, Germany) was applied for HSI analysis. For the ICG images, Image J (Wayne Rasband, National Institutes of Health, Bethesda, MD, USA) and Vision Sense 1.52 (Medtronic GmbH, Meerbusch, Germany) were chosen. The reference point for the distance measurements was the blue mark from the subjective transection line. Proximal (central) deviations to this line were annotated with (−), while distal (peripheral) deviations with (+). Both imaging methods resemble different measurement scales. Currently, there are no cut-off values or thresholds established to define adequate and poorly perfused tissue. We defined good tissue perfusion in HSI as >75% StO_2_ (Figure 1a). The ROI (region of interest) consisted of ten markers—4 mm in diameter—along an imaginary line from central to peripheral on the gastric tip. In FI, we defined the border zone at a 50% decline in the intensity from maximum perfusion. Further determination of the border zone was analogous to HSI measurement. The definition of the anastomotic site according to the data acquired by HSI and FI-ICG was compared with our subjective prediction that was marked by the blue line intraoperatively (Figure 3). The estimated borderline was considered as an upper border of the circular anastomosis, revealing that the anastomosis would be completed in the well-perfused area.

### 2.7. Follow-Up and Endpoints

The primary endpoint was the determination of the “ideal” anastomotic site during oncologic esophagectomy by both imaging modalities. The secondary endpoint was postoperative AL. After surgery, the patients were followed up for 14 days or until their discharge. A routine oral methylene blue swallow test was performed on the 6th postoperative day. According to our internal guidelines, if this had turned positive (via the intraoperatively placed chest tube), an upper gastrointestinal (GI) endoscopy and an oral contrast-enhanced computed tomography (CT) usually had been carried out consecutively. AL was defined as “full thickness GI defect involving esophagus, anastomosis, staple line, or conduit, irrespective of presentation or method of identification”, according to the “Esophageal Complications Consensus Group” (ECCG) 2015 [30]. Further postoperative complications were assigned according to the Clavien–Dindo Classification (CDC) [31].

### 2.8. Statistical Analysis

Mean and median values of the measurements were calculated in Excel. The Kolmogorov–Smirnov test showed a normal distribution. A paired *t*-test was performed in order to detect basic mean differences between the HSI and ICG measurements. Additionally, the grade of agreement between the imaging methods was calculated with an “Intraclass Correlation Coefficient” (ICC). A *p*-value of <0.05 was considered statistically significant for all procedures.

## 3. Results

### 3.1. Patients’ Characteristics

All included patients underwent ischemic preconditioning of the stomach three to six days before gastric conduit formation (median = 5 days; range = 3–6 days). The cohort had a median age of 63 (range: 48–78) years and consisted of twelve males and one female. Eight patients had adenocarcinoma (61.5%), three had squamous cell carcinoma (23.0%), one had an adenosquamous carcinoma (7.7%), and another patient had a mixed adenoneuroendocrine carcinoma (MANEC) (7.7%). Median body mass index (BMI) was 25 (18–47) kg/m^2^. Neoadjuvant radiochemotherapy according to the CROSS-protocol (ChemoRadiotherapy for esophageal cancer followed by Surgery Study)/perioperative chemotherapy with FLOT (fluorouracil–leucovorin–oxaliplatin–docetaxel) was performed in twelve patients (93.3%). The mean operation time of the thoracic stage was 4 h 11 min (SD = ±51 min). The patients’ characteristics are presented in Table 1.

### 3.2. Intraoperative HSI and FI-ICG of the ROI (Gastric Tip)

We were able to perform HSI and FI-ICG in all the included patients (*n* = 13) of this pilot study. In cases of differences between imaging results, the more central measurement was chosen for anastomosis for safety reasons. Sufficient perfusion of the ROI was detected in ten patients (76.9%) peripheral to the marked line. In these, an additional distance of median 14.6 mm (SD = ±11.3 mm) of the well-perfused area located peripheral to the landmark was discovered. The range was 2.4–36.0 mm. This distance was 22.4 mm (SD = ±22.2 mm) according to the quantification of the FI-ICG signal based on the intensity of the fluorescence and 16.6 mm (SD = ±17.1 mm) according to the HSI measurements. In five of the patients (38.5%), the upper border of the anastomosis was adapted peripherally to the marked blue line. The range of the corrected distances in these five patients was 10–25 mm, with a median of 20 mm. No further adaption at the anastomotic site was necessary in the other five patients. In three patients (23.1%), the upper border of the anastomotic site was equal to the subjectively marked blue line (Figure 1). There was no central adaption to the blue line according to HSI and FI-ICG measurements. There were no significant differences between these two modalities. The minimum and maximum differences are presented in Figure 3. Therefore, in all 13 patients, a well-perfused area of about 9.6 mm (SD = ±14.2) above the end of the right gastroepiploic arcade was detected. Thus, a well-perfused and tension-free anastomosis was made in all of the patients included in this study.

The Kolmogorov–Smirnoff test indicated a normal distribution for the HSI and FI-ICG measurements. Therefore, paired *t*-tests were conducted. There were no significant differences in distances between the HSI and FI-ICG measurements (mean 3.6 mm, SD = ±17.0 mm; *p* = 0.46). Further contemplations with intraclass correlation (ICC) for average measurement (0.66) showed a substantial agreement of the two imaging methods. The correlation between the HSI and ICG distance measurement was *r* = 0.48.

### 3.3. Postoperative Findings and Follow-Up

There were no complications due to ICG injection. None of the patients developed AL in the postoperative course. Postoperative complications grade I according to the CDC were found in three patients (23%): the first one with fever of unknown origin and the other one with pleural effusion and atelectasis. The third patient had dyspnea and chills, which resulted in computer tomography (CT) scan on day six after surgery, but without conspicuous results and with spontaneous recovery. Postoperative complications grade II were found in one patient (7.7%) with pneumonia and resulted in treatment with antibiotics (Cefotaxim i.v.). Another patient (7.7%) had postoperative complication grade IV, which resulted in reintubation due to pneumonia, delirium, and sepsis. Consecutively, treatment with piperacillin/tazobactam was completed, and the patient recovered.

## 4. Discussion

AL is one of the most serious postoperative complications after oncologic esophagectomy, associated with a high rate of associated mortality. Sufficient perfusion of the gastric conduit, especially of the anastomotic site, is crucial for undisturbed healing. The best method of exactly estimating the perfusion of the anastomosis during esophagectomy is still controversial.

In this pilot study, we focused on the comparison of the two most promising intraoperative imaging methods for perfusion assessment. According to our knowledge, this is the first study to generate objective criteria in evaluating the anastomotic site during esophagectomy by means of HSI and FI-ICG simultaneously. A well-perfused and tension-free anastomosis could be performed in all patients included in this study. Fortunately, in ten out of thirteen patients (76.9%), the combination of the imaging methods identified a more peripheral perfusion border zone than the surgeon’s subjective evaluation. This could be explained through a rich vascular network of the gastric wall intramurally, which can usually not be visualized without imaging methods. So far, only a few clinical studies have attempted to quantify the FI-ICG signal based on the time course after dye injection, and no gold standard has been established to date. Kumagai et al. described a sufficient blood flow of the gastric tube if the anastomosis was made in the area, where less than 60 s were needed for FI-ICG enhancement [12]. Complementarily to Nerup et al., who convincingly demonstrated that subjective ICG measurements slightly overestimate perfusion, our pilot study points in the same direction, with FI-ICG measurements being on average 3.6 mm more peripheral than the HSI measurements [32]. These findings could potentially be explained by capillary diffusion of ICG molecules—over time—into ischemic areas, as Diana et al. have hypothesized [33]. Nevertheless, the patient cohort is too small to make further conclusions. Lütken et al. evaluated different ways for quantification of FI-ICG and concluded that a combination of timing and inflow intensity reflect clinical outcomes at best [34]. To date, there is no consensus on the best quantification method for FI-ICG, because it is highly complex, results are conflicting, and no cut-off values have been established yet [34]. During the last years, our group has clearly demonstrated the impact of using HSI in evaluating the perfusion of the gastric tube during esophagectomy [17,22]. However, larger studies are still missing, and this is the first pilot study to measure clinical outcomes in HSI and simultaneous FI-ICG perfusion assessment in oncologic esophagectomy. Jansen-Winkeln et al. already demonstrated that both imaging methods are comparable to each other in colorectal anastomotic assessment [19]. This is consistent with the findings in our current study, as we did not reveal significant differences between both modalities (*p* = 0.46). Köhler et al. proved that HSI is suitable to examine ischemic conditioning effects of the gastric conduit [17]. Barberio et al. in an animal trial transferred the HSI technology into virtual reality (HYPER), which overcomes the limitation that the information is given on a screen without the corresponding spatial data [18]. The major limitation of this pilot study is its small sample size. Furthermore, we have chosen subjective values for HSI and FI-ICG border zone determination due to lacking the established thresholds. The subjective FI-ICG was chosen due to the evidence gap that one specific quantification method is superior to the others and FI-ICG without quantification is the most widespread method. Another limitation is that the time duration between HSI and FI-ICG measurements was not standardized. This is due to multiple reasons, such as communication with anesthesiologists, varying preparation times of the dye, and technical issues. This could potentially have led to altered perfusion over time between the HSI and the FI-ICG measurements. Furthermore, it should be mentioned that our measurements were performed only before the formation of the intrathoracic anastomosis, the reason being that the HSI camera system—until just recently—had been designed for an open surgical environment. After the stapler connection of the gastric tube and the remaining esophagus, the finalized anastomosis deep in the thoracic cavity below the mini-thoracotomy had been unobtainable for the HSI system. Factors that led to differences in the measurement results were probably mainly handling related and emphasize the importance of measurement standardization. Finally, we demonstrated that HSI and FI-ICG are comparable with complementary value added and that it is easily practicable to combine both methods in the clinical routine of oncologic esophagectomy. In accordance with our previous research, this study indicates that HSI and FI-ICG are both reliable predictors of adequate perfusion, although intra- and post-operative cardiovascular parameters, volume and vasopressor management might be additional relevant factors affecting this key parameter and, thus, the healing process of the anastomosis. Both imaging tools have advantages and disadvantages, but as we have shown, simultaneous usage is feasible, time-effective, and leads to satisfying results regarding anastomotic leaks. Future research will focus on the differentiated outcomes of both imaging parameters in larger cohorts. If HSI proves its reliability in larger multi-center studies, it is imaginable that HSI will supersede FI-ICG, based on its easier handling, the possibility for repetitive measurements, lower costs, non-invasiveness, and non-existing contraindications. An endoscopic/minimally invasive system for HSI measurement is already in use in our clinic and will broaden the spectrum of image-guided surgery [25].

## 5. Conclusions

Our data clearly support the prior findings that HSI and FI-ICG are comparable imaging methods and can be used complementarily. No anastomotic leak occurred during this pilot study. Both novel imaging modalities are rapidly evolving and are, for now, superior to other perfusion assessments or surgeons’ subjective evaluations. Future research will be needed in order to determine which intraoperative image tools will have the potential to become the new gold standard in oncologic esophagectomy.

## Figures and Tables

**Table 1:** Preoperative findings and demographic data.

Variables	Number of Cases (%)
**Sex**	
male	12 (92.3)
female	1 (7.7)
**ASA** *	
I	1 (7.7)
II	7 (53.8)
III	4 (30.8)
IV	1 (7.7)
**Neoadjuvant** **therapy**	
none	1 (7.7)
chemotherapy (FLOT)	7 (53.8)
radiochemotherapy (CROSS) **	5 (38.5)
**Histopathologic** **entity**	
squamous cell carcinoma	3 (23.0)
adenocarcinoma	8 (61.5)
MANEC, adenosquamous	2 (15.4)
**Risk** **factors**	
smoking	8 (61.5)
COPD *** Gold II	6 (46.2)
diabetes mellitus type II	1 (7.7)
arterial hypertension	5 (38.5)
pulmonary embolism	1 (7.7)
renal insufficiency	1 (7.7)

* ASA: American Society of Anesthesiologists-classification; ** ChemoRadiotherapy for esophageal cancer followed by Surgery Study)/perioperative chemotherapy with FLOT (fluorouracil–leucovorin–oxaliplatin–docetaxel; *** COPD: Chronic Obstructive Pulmonary Disease.

**Table 2:** Major steps during a standard two-stage esophagectomy with ischemic conditioning of the stomach.

Step 1 Laparoscopic/Robotic Gastrolysis	Step 2 (after 3–6 Days) Ivor Lewis Esophagectomy
- stomach mobilization and partial devascularization of the stomach (left gastro-epiploic, short gastric and left gastric vessels)	- relaparoscopy and gastric tube formation - right-sided transthoracic esophagectomy, and systematic lymphadenectomy (double lumen intubation)
- preparation of the omentum flap (fundus/corpus)	- gastric pull-up - intraoperative imaging with HSI and FI-ICG
- abdominal 2–lymphadenectomy	- high-intrathoracic esophago-gastric anastomosis with a circular stapler (25 or 28 mm)
	- omentum flap between trachea and anastomosis

## Data Availability

The data presented in this study are available in this article.
